# Ni_2_P nanocrystals embedded Ni-MOF nanosheets supported on nickel foam as bifunctional electrocatalyst for urea electrolysis

**DOI:** 10.1038/s41598-021-00776-8

**Published:** 2021-11-01

**Authors:** Haitao Wang, Haiyan Zou, Yingying Liu, Zhenglong Liu, Wenshuang Sun, Kunyi Andrew Lin, Tielong Li, Shuangjiang Luo

**Affiliations:** 1 Tianjin Key Laboratory of Environmental Technology for Complex Trans-Media Pollution, MOE Key Laboratory of Pollution Processes and Environmental Criteria, College of Environmental Science and Engineering, Nankai University, Tianjin, 300350 China; 2 Department of Environmental Engineering & Innovation and Development Center of Sustainable Agriculture & Research Center of Sustainable Energy and Nanotechnology, National Chung Hsing University, 250 Kuo-Kuang Road, Taichung, Taiwan; 3 Institute of Process Engineering, Chinese Academy of Sciences, Beijing, 100190 China

**Keywords:** Energy science and technology, Materials science

## Abstract

It’s highly desired but challenging to synthesize self-supporting nanohybrid made of conductive nanoparticles with metal organic framework (MOF) materials for the application in the electrochemical field. In this work, we report the preparation of Ni_2_P embedded Ni-MOF nanosheets supported on nickel foam through partial phosphidation (Ni_2_P@Ni-MOF/NF). The self-supporting Ni_2_P@Ni-MOF/NF was directly tested as electrode for urea electrolysis. When served as anode for urea oxidation reaction (UOR), it only demands 1.41 V (vs RHE) to deliver a current of 100 mA cm^−2^. And the overpotential of Ni_2_P@Ni-MOF/NF to reach 10 mA cm^−2^ for hydrogen evolution reaction HER was only 66 mV, remarkably lower than Ni_2_P/NF (133 mV). The exceptional electrochemical performance was attributed to the unique structure of Ni_2_P@Ni-MOF and the well exposed surface of Ni_2_P. Furthermore, the Ni_2_P@Ni-MOF/NF demonstrated outstanding longevity for both HER and UOR. The electrolyzer constructed with Ni_2_P@Ni-MOF/NF as bifunctional electrode can attain a current density of 100 mA cm^−2^ at a cell voltage as low as 1.65 V. Our work provides new insights for prepare MOF based nanohydrid for electrochemical application.

## Introduction

In recent years, the application of metal organic frameworks (MOFs) in the electrochemical field, especially electrocatalysis, has attracted considerable interest. The major appeals of the electrochemical application of MOFs are their large surface area, well-defined pores and tunable chemical composition^1,2^. However, the direct use of MOFs as electrode is often limited due to the poor intrinsic conductivity. So, MOFs are more often used as precursors to prepare advanced electrocatalysts through carbonization at high temperature^3^. The organic ligands in the MOFs are pyrolyzed to form graphitized/amorphous carbon matrix that could serve as freeway for electron flow. As a result, the MOFs-derived material through carbonization exhibited enhanced electrochemical activity for both hydrogen evolution reaction (HER) and oxygen evolution reaction (OER) due to the dramatically increased conductivity^4^. The electrocatalytic performance of the carbonized material can be further boosted by phosphidation^5^. However, the fascinating pore structure of MOFs is largely destroyed during the heat treatment, vanishing the advantages associated with the well-defined pore structure as well as high specific surface area^6^.

In recent years, MOF based nanohybrids prepared by integration of nanoparticles with MOF have attracted considerable attention^7,8^. Beside utilizing the super adsorption capability of MOF, such integration strategy could tuning the microenvironment of the nanoparticles to improve their catalytic activity^8^. Nevertheless, it is still very challenging to synthesize self-supporting MOF-nanoparticles nanohybrids, which is highly desired for the application in the electrochemical field.

On the other hand, OER is a thermodynamically sluggish multi-electron process with large over-potential, leading to energy consumption surge for electrochemical hydrogen production^9^. Moreover, it is a formidable challenge to completely avoid the formation of explosive H_2_/O_2_ mixtures during water electrolysis^10^. Replacing OER with alternative electrochemical oxidation reaction offers an effective way to address these drawbacks^11^. So far, urea, methanol and hydrazine electrochemical oxidation have been explored as alternative anode reaction^12–16^. Compared with others, electrochemical oxidation of urea (UOR) has multiple advantages: (1) urea is a widely available commodity with merits such as non-toxic, high soluble in water, non-flammable, high energy density, easy storage and transportation; (2) the end products of UOR are harmless gases CO_2_ and N_2_; (3) Furthermore, it is feasible to extract energy from urea-rich wastewater such as human/animal urine, effluents discharged from urea synthesis factory^17–19^. Since the application of earth-abundant nickel as electrocatalyst for UOR was first reported by G. Botte et al^20^, various nickel-containing nickel materials such as nickel oxides, sulfides, phosphides and alloys have been extensively explored as electrocatalyst for UOR^21–35^.

Herein, we report a feasible way to prepare Ni_2_P nanocrystals embedded Ni(BDC)(DMF) MOF supported on nickel foam (Ni_2_P@Ni-MOF/NF) through a direct phosphidation process. During the phoshidation process, part of the nickel atoms in the Ni-MOF transformed into Ni_2_P nanoparticles. The self-supporting Ni_2_P@Ni-MOF/NF was directly used as electrode for HER and UOR without the use of any polymer binders. The overpotential was only 66 mV to drive HER at a current density of 10 mA cm^−2^. To drive UOR at a current density of 100 mA cm^−2^, it only needs a potential as low as 1.41 V (vs RHE). The superior HER and UOR performance of the Ni_2_P@Ni-MOF/NF was attributed to the enhanced conductivity, fast release of the gases bubbles from the surfaces of the electrode as well as the tuning of the microenvironment of Ni_2_P nanoparticls by Ni-MOF. The electrolyzer constructed with Ni_2_P@Ni-MOF/NF as both bifunctional electrode could deliver a current density of 100 mA cm^−2^ in 1 M NaOH with the presence of 0.33 M urea at 1.65 V, which was 0.26 V lower than water electrolysis.

## Results and discussion

### Materials characterization

**Figure 1:**
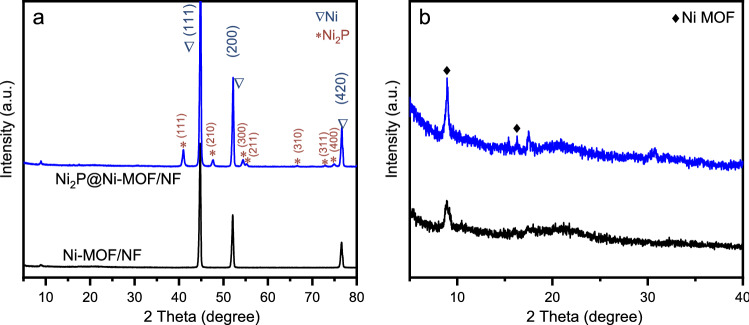
(**a**) Powder XRD pattern of Ni-MOF/NF and Ni_2_P@Ni-MOF/NF and (**b**) enlarged XRD pattern on an expanded y-axis scale.

To synthesize the Ni_2_P@Ni-MOF/NF electrode, the Ni(BDC)(DMF) MOF was first grown on NF by a solvothermal method at 140 °C and the sample was labelled as Ni-MOF/NF. Afterwards, the Ni-MOF was converted to Ni_2_P@Ni-MOF by a direct phosphidation process carried out at 300 °C. Powder X-ray diffraction (XRD) was first employed to get the composition and phase of the Ni-MOF/NF. Two sharp diffraction peaks at 44.9° and 52.2° were observed in the XRD pattern of Ni-MOF/NF (Fig. 1a), corresponding to the (111) and (200) planes of face-centered cubic nickel (JCPDS No. 01–1260). Apparently, the diffraction peaks of Ni-MOF were relatively weak. Enlarged XRD patterns on an expanded y-axis scale was provided in Fig. 1b for clarity. The diffraction peak at 8.9° and 17.5° was assigned to Ni-MOF (CCDC No. 638866). After phosphidation process, four new peaks emerged at 40.9°, 47.6°, 54.4° and 55.1°, which were attributed to the (111), (210), (300) and (211) planes of hexagonal Ni_2_P (JCPDS No.03–0953). The diffraction peaks at 8.9° and 17.5° associated with Ni-MOF did not disappear, indicating only part of the Ni-MOF has been converted to Ni_2_P. The thermogravimetric analysis curve shown in Fig. S1 confirmed that 300 °C was not high enough to cause the decomposition of Ni-MOF.

The FTIR spectra of the nanosheets before and after phosphidation are shown in Fig. S2. Overall, the absorption bands of nanosheets became weaker after phosphidation. Nevertheless, the FTIR result verified the partial preserve of Ni-MOF. The absorption band around 3444 cm^−1^ was assigned to the stretching vibration of NH. The asymmetric and symmetric stretching modes of C = O appeared at 1582 and 1369 cm^−1^, respectively^36,37^. The band at 1087 was attributed to the stretching vibration of C–O of carboxylic acid group. And narrow bands at 1025 and 748 cm^−1^ were attributed to δ(C–H) and γ(C–H) vibration of aromatic rings, respectively.

**Figure 2:**
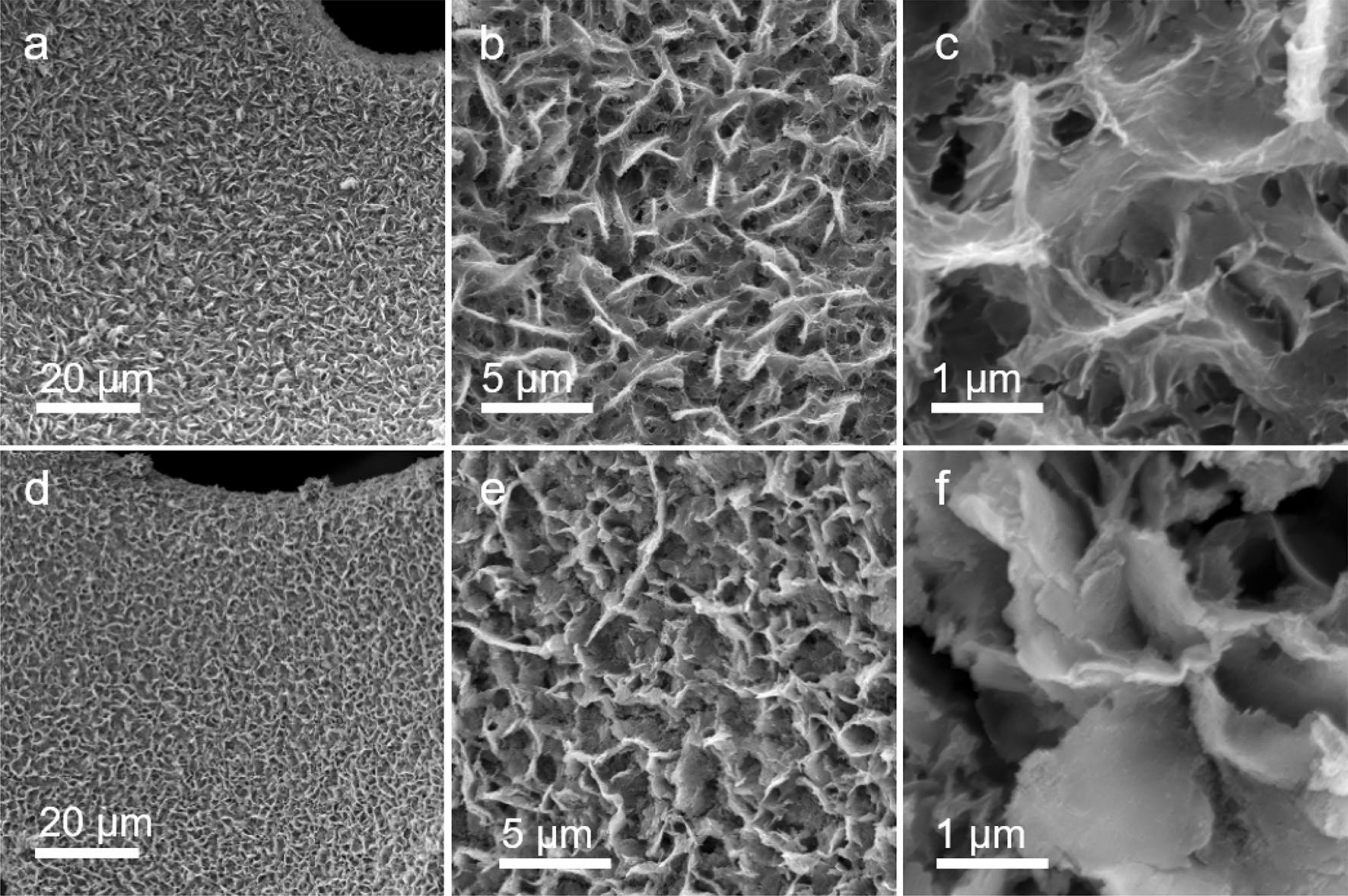
SEM image of Ni-MOF/NF (**a**–**c**) and Ni_2_P@Ni-MOF/NF (**d**, **e**) under different scales.

As revealed by the SEM characterization (Fig. S3), the Ni foam exhibited a highly open pore structure with pore size around several hundred microns. The high resolution SEM image revealed that the nickel skeleton was composed of relatively smooth large compact Ni grains. After Ni-MOF growth, the surfaces of Ni foam were uniformly covered with 2D nanosheets (Fig. 2). The Ni-MOF nanosheets exhibited a leaf-like morphology with lateral size 4–7 microns, which interlaced with each other to form a nest-like structure. The high resolution SEM image shown in Fig. 2c disclosed the fine vein-like structure on the surfaces of the as-grown Ni-MOF. After phosphidation process, the skeleton of Ni foam was still uniformly covered with nanosheets (Fig. 2d). But compared to Ni-MOF/NF sample, the population of the nanosheets was apparently decreased. As it can be clearly seen in the high resolution SEM images (Fig. 2e, f), the fine vein-like structures disappeared after phosphidation and the surfaces of the 2D nanosheets became smoother. Nevertheless, the sample still exhibited nest-like morphology composed of 2D nanosheets. The successfully incorporation of P elements through phosphidation was confirmed by the energy-dispersive X-ray (EDX) spectrum (Fig. S4).

**Figure 3:**
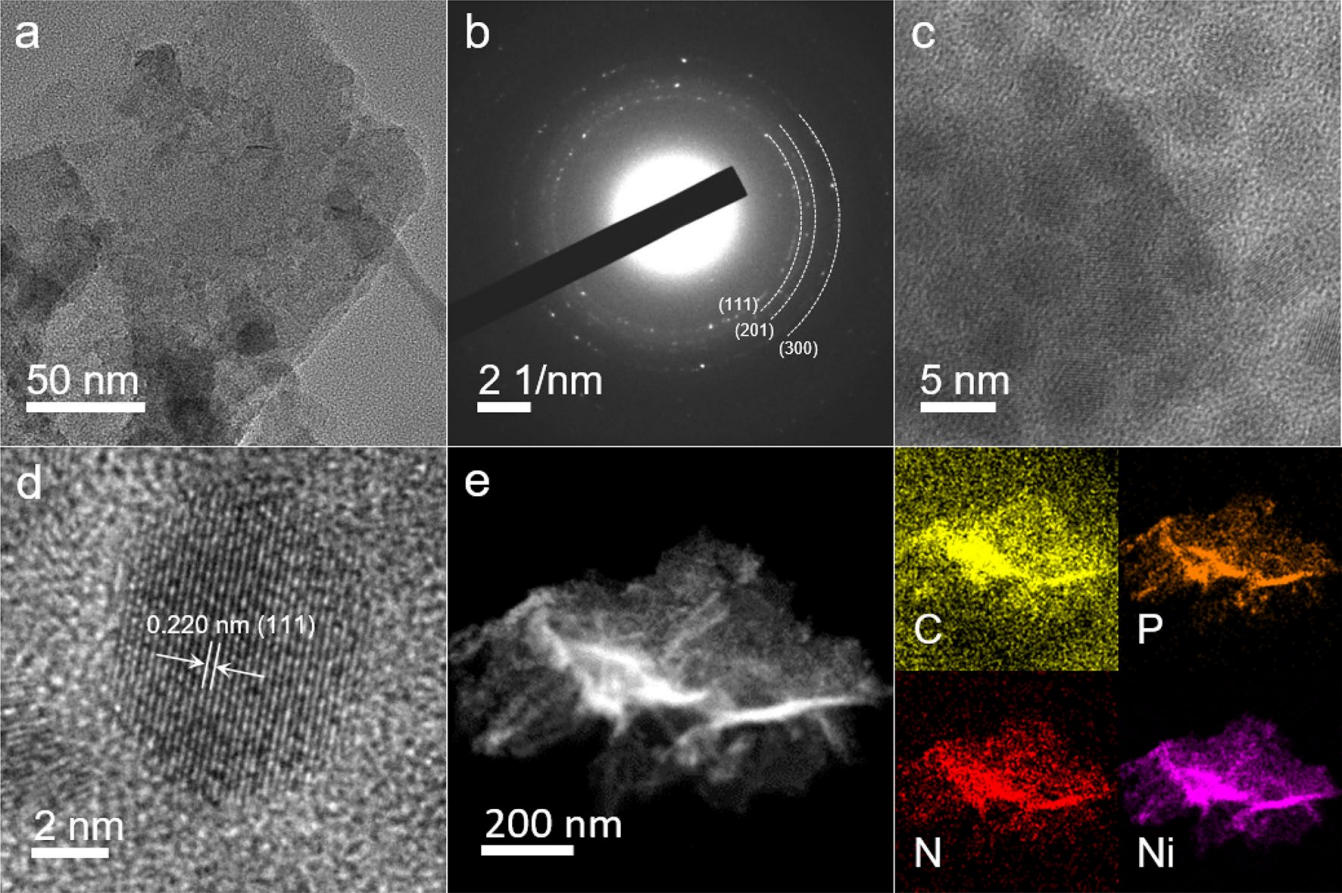
(**a**) TEM image, (**b**) SAED, (**c**, **d**) HRTEM image, and (**e**) elemental mapping image of Ni_2_P@Ni-MOF.

For TEM characterization, the Ni_2_P@Ni-MOF nanosheets were scraped off the nickel foam and dispersed in ethanol with the help of vortex shaking instead of sonication to minimize the damage. The TEM image shown in Fig. 3a reveals the 2 dimensional nature of the Ni_2_P@Ni-MOF sample. The selective area electron diffraction (SAED) pattern displayed rings with bright spots, indicating the polycrystalline nature of the sample (Fig. 3b). High resolution TEM (HR-TEM) image revealed that the surface of the nanosheet was decorated with nearly monodispersed nanoperticles with size around 8 nm (Fig. 3c). The formation of Ni_2_P nanoparticles was limited by the available nickel atoms in the MOF nanosheets, similar to the formation of metal or metal oxide nanoparticles in layer double hydroxide^38^. The HR-TEM image of a single nanoparticle shows distinct lattice fringes with interplanar d-spacing determined to be 0.22 nm (Fig. 3d), which was attributed to the (111) plane of Ni_2_P. According to the elemental mapping image, the Ni, P, C and N elements were uniformly distributed in the Ni_2_P@Ni-MOF nanosheet (Fig. 3e). The atomic ratio of Ni:P was ~ 5.6:1, proving the partial transformation of the Ni atoms in the Ni-MOF to Ni_2_P.

**Figure 4:**
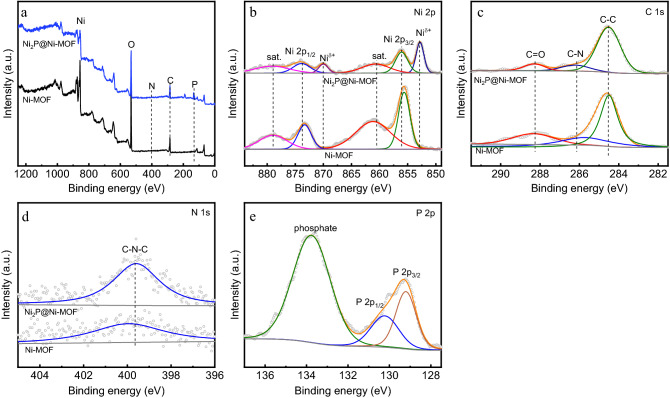
(**a**) XPS survey spectra of Ni-MOF/NF and Ni_2_P@Ni-MOF/NF and high resolution XPS spectra of (**b**) Ni 2p, (**c**) C 1 s, (**d**) N 1 s and (**e**) P 2p. All of the spectra were calibrated by C1s peak located at 284.8 eV.

XPS was performed employed to investigate the surface chemical composition and electronic state of elements of the nanosheets before and after phosphidation. As shown in the XPS survey spectra (Fig. 4a), besides the Ni, C and N elements, new signal peak positioned at 133 eV corresponding to P element after phosphidation process. The high resolution Ni 2p XPS spectrum of Ni-MOF displayed four prominent peaks (Fig. 4b), the two peaks positioned at 855.6 and 873.5 eV are assigned to Ni 2p_3/2_ and Ni 2p_1/2_, respectively^39,40^. The other two peaks positioned at 861.8 and 878.2 eV are the corresponding satellite peaks of Ni 2p_3/2_ and Ni 2p_1/2_, respectively. After phosphidation, two new peaks corresponding to reduced Ni^*δ*+^ emerged at 852.8 and 870.0 eV^41^. Meantime, both the Ni 2p_3/2_ and Ni 2p_1/2_ peaks shifted to higher binding energy, suggesting the charge transfer from nickel to phosphorus atoms. The high resolution C1s XPS spectrum of Ni-MOF nanosheets displays two main peaks and the one positioned at 288.3 eV is assigned to C=O (Fig. 4c)^42^. The other peak positioned at 284.5 eV could be deconvoluted into two peaks corresponding to C–C (284.5 eV) and C–N (285.0 eV), respectively. After phosphidation, the peak related to C–N shifted 0.4 eV to higher binding energy, suggesting the charge transfer from carbon to neighboring atoms. The N 1s XPS spectrum of Ni-MOF nanosheets only displays one peak at 399.9 eV assigned to C–N–C from DMF (Fig. 4d). After phosphidation, the peak shifted slightly to lower binding energy to 399.6 eV, demonstrating the charge transfer from neighboring atoms to N. The N 1s peak could not be deconvoluted, suggesting that no graphitized nitrogen formed after phosphidation process. That was reasonable since the temperature of phosphidation process was too low to induce graphitization. The high resolution P 2p XPS spectrum exhibits two main characteristic peaks positioned at 129.3 and 134.7 eV (Fig. 4e), which are assigned to P-Ni and oxidized phosphate species^43,44^.

### UOR performance

**Figure 5:**
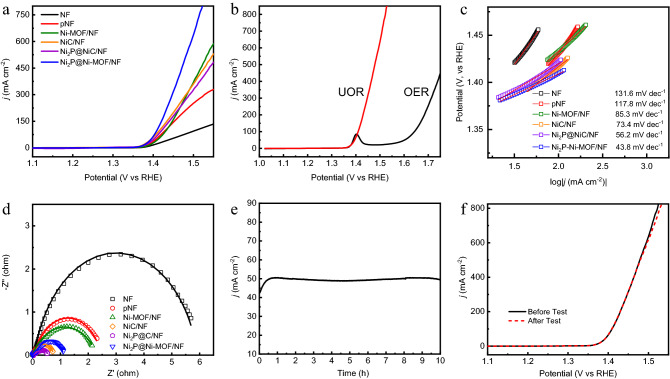
(**a**) LSV curves of Ni_2_P@Ni-MOF/NF, Ni_2_P@NiC/NF, NiC/NF, Ni-MOF/NF, pNF and NF in 1.0 M NaOH with 0.33 M urea. (**b**) LSV curve of Ni_2_P@Ni-MOF/NF in 1.0 M NaOH with or without 0.33 M urea. (**c**) The corresponding Tafel plots and (**d**) the Nyquist plots of various electrodes. (**e**) Chronoamperometric (i-t) curve of Ni_2_P@Ni-MOF/NF for 10 h. (**f**) LSV curves of Ni_2_P@Ni-MOF/NF before and after 10 h chronoamperometry test.

The UOR performance of various electrodes were evaluated by using LSV technique in a mixed solution containing 1 M NaOH and 0.33 M urea. As shown in Fig. 5a, the initial oxidation potentials of UOR for various electrodes were almost the same, 1.36 V (vs RHE). This result suggested that the active species for UOR of various electrode were the same as reported in previous study^45^. The Ni_2_P@Ni-MOF/NF electrode demonstrated the best UOR activity, followed by Ni-MOF/NF. The potential to drive the UOR at 100 mA/cm^2^ is only 1.41 V, which is among the best when compared with the literature data (Table S1). The linear increase of the current density above 1.38 V (vs RHE) indicated the readily detachment of gases bubbles from the surfaces of the electrode^45^. The UOR performances of NiC/NF and Ni_2_P@NiC/NF were inferior to Ni-MOF/NF, which may be attributed to the encapsulation of nickel species by carbon.

The current density reported in this work was normalized to the projected area of the electrode, which could not reflect the intrinsic catalytic activity of the material^46^. However, it was impossible to accurately determine the loading amount of the active material on the electrode. So, we tried to normalize the current density to the electrochemically active surface area (ECSA), which was regarded to be directly correlated with the active sites for electrochemical reaction^47^. The ECSA of the electrode could be calculated by using the following equation,

1 $$ {\text{ECSA}} = {\text{C}}_{{{\text{dl}}}} {\text{/C}}_{{\text{s}}} $$

where C_dl_ is the double-layer capacitance of the electrode, C_s_ is the specific capacitance of the material. The C_dl_s of various electrodes were determined from the CV curve obtained in a non-Faradic region (0.1–0.2 V) in 1 M NaOH solution (Fig. S5 and Fig. S6a). Since C_s_s of various materials are unknown for, the ECSAs cannot be determined. The current density was therefore normalized to C_dl_ by assuming that all materials have the same C_s_ (Fig. S6b). Obviously, the Ni-MOF/NF electrode had the best performance, followed by Ni_2_P@Ni-MOF/NF.

Since OER was the competitive reaction with UOR at higher potential, the OER performance was also investigated (Fig. 5b). An obvious anodic peak corresponding of the oxidation of Ni^2+^ to Ni^3+^ was observed in the polarization curve of OER at the potential of 1.40 V (vs RHE). By comparing the LSV curve of UOR to OER, it is clear that the oxidation of urea occurs upon the formation of Ni^3+^ species, demonstrating that UOR happens readily than OER. In sharp contrast to UOR, OER occurs at a relatively high potential (1.6 V vs RHE) after the completion of the oxidation of Ni^2+^ to Ni^3+^, revealing that the real catalyst for OER is NiOOH^48^. It is noteworthy that the current density for UOR reached 850 mA/cm^2^ before the occurring of OER, which is the highest ever reported to the best of our knowledge. Tafel analyses were conducted to compare the UOR kinetics of various catalysts. As shown in Fig. 5c*,* the Ni_2_P@Ni-MOF/NF electrode exhibited the lowest Tafel slope of 43.8 mV/dec, significantly smaller than other catalysts. The results indicated that the UOR with Ni_2_P@Ni-MOF/NF electrode occurred at faster kinetics than other electrodes.

The charge transfer resistance is a critical influential parameter for electrochemical reaction, which is commonly negative correlated with conductivity of the electrode. In order to evaluate the charge transfer resistance during UOR, electrochemical impedance (EIS) tests were carried out at 1.42 V. The best fitting was obtained using the equivalent circuit shown in Fig. S7. As shown in Fig. 5d, all of the Nyquist plots exhibited a semicircle shape. The smaller diameter of the semicircle, the lower the charge transfer resistance. Obviously, the Ni_2_P@Ni-MOF/NF electrode is not the smallest, which is slight larger than that of Ni_2_P@NiC/NF and NiC/NF. The fitting results indicated that the Ni_2_P@NiC/NF had the smallest charge transfer resistance (Table S2), clearly demonstrating that high temperature carbonization and phosphidation was the best way to enhance the conductivity. On the other hand, the results also indicate that conductivity is not the only determinant factor for UOR.

Chronoamperometry test was conducted at 1.4 V (vs RHE) to investigate the durability of Ni_2_P@Ni-MOF/NF electrode. As shown in Fig. 5e, the current density gradually increased from 42 to 50 mA cm^−2^ in the early stage, which may be attributed to the activation of electrode. At the end of the stability test, the current density declined by only 1.9%, demonstrating the superior stability of the electrode. UOR involves gas evolution, the gas bubbles formed on the surface of the electrode need to be released promptly to avoid the blocking of the active sites^49^. The blocking effect will be exacerbated at constant applied potential, which leads drastic fluctuation of the current density^50^. It can be clearly seen from Fig. 5e that the current density fluctuation was negligible, suggesting the swiftly gases bubbles detachment from the electrode’s surface. This fast detachment of gases bubbles was credited to the surface properties of the electrode and the porous structure of the nickel foam. The interconnected macroporous structure of nickel foam allows fast detachment of tiny gas bubbles, enabling it an ideal platform to prepare advanced electrodes for gas evolution reaction^51,52^. The LSV test was conducted again after durability test. As shown in Fig. 5f, the LSV curve overlapped with that collected before durability test. The results verified the excellent longevity of the electrode for UOR.

**Figure 6:**
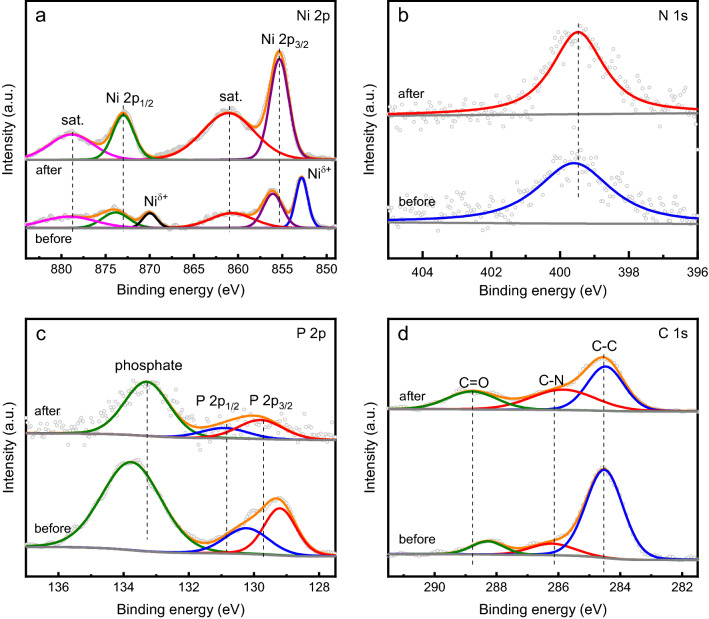
High resolution XPS spectra of (**a**) Ni 2p, (**b**) N 1 s, (**c**) P 2p and (**d**) C 1s in Ni_2_P@Ni-MOF/NF after durability test.

The Ni_2_P@Ni-MOF/NF electrode after stability test was first characterized by using XPS. As shown in Fig. 6a, the XPS peaks corresponding to the Ni^*δ*+^ species decreased dramatically after test. And the XPS peaks related to Ni 2p shifted to higher binding energy level. The result indicated the oxidation of Ni species to higher valance state in the material. No change was observed for N 1s peak (Fig. 6b). The P 2p peak shifted to higher binding energy level, indicating the charge transfer form P atoms to neighboring atoms (Fig. 6c). The shifting to high energy level was also observed for the XPS peak corresponding to C=O (Fig. 6d). Overall, the XPS result indicated the occurrence of the oxidation of surface elements of the electrode. The morphology of the catalyst was further characterized by using SEM and TEM. As shown in Fig. S8a, no apparent morphology deterioration was found after test. And the lattice fringes of Ni_2_P could also be resolved as shown in the TEM image (Fig. S8b). The elemental mapping confirmed that the main components of the catalyst was still the same (Fig. S8c).

### HER performance

**Figure 7:**
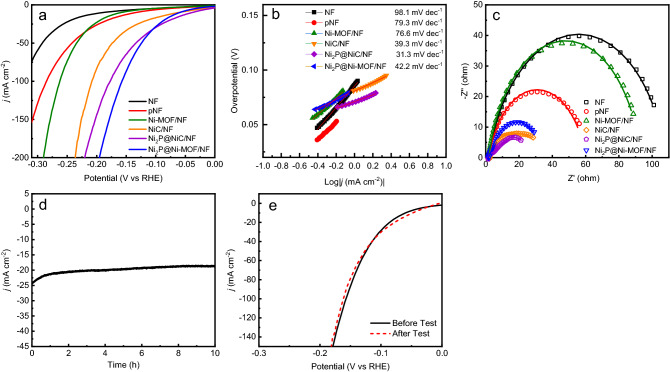
(**a**) LSV curves, (**b**) the corresponding Tafel plots and (**c**) the Nyquist plots of Ni_2_P@Ni-MOF/NF, Ni_2_P@NiC/NF, NiC/NF, Ni-MOF/NF, pNF and NF, (**d**) Chronoamperometric (i-t) curve of Ni_2_P@Ni-MOF/NF for 10 h. (**e**) LSV curves of Ni_2_P@Ni-MOF/NF before and after durability test. The electrolyte was 1.0 M NaOH with 0.33 M urea.

Nickel phosphides such as Ni_2_P and Ni_5_P_4_ have been extensively explored as electrocatalyst for HER^53,54^. So, the HER performance of Ni_2_P@Ni-MOF/NF electrode was evaluated by using LSV technique in 1 M NaOH containing 0.33 M urea. From Fig. 7a, Ni_2_P@Ni-MOF/NF electrode had apparent better electrocatalytic activity for HER compared with other electrodes. The overpotential at current density of 10 mA cm^−2^ (η_10_) follows the order: Ni_2_P@NiC/NF (57 mV) < Ni_2_P@Ni-MOF/NF (6 mV) < NiC/NF (78 mV) < pNF (144 mV) < Ni-MOF/NF (165 mV) < NF (200 mV). The result clearly manifested that phosphidation and carbonization could dramatically boost the HER performance of the Ni-MOF. Although the η_10_ of Ni_2_P@Ni-MOF/NF was larger than that of Ni_2_P@NiC/NF, the performance of electrode at large current density was inferior to the latter one. Moreover, the mechanical properties of Ni_2_P@NiC/NF were very poor, material fragments kept falling off the electrode during the electrochemical test. The HER activity of the Ni_2_P@Ni-MOF/NF electrode, in terms of the η_10_ value, is lower than many state-of-the-art NF based HER electrodes such as NiFe-MOF/ NF (134 mV) and Ni_2_P/Ni/NF (98 mV) (Table S3)^18,55–60^. The Tafel slope of Ni_2_P@Ni-MOF/NF was 42.2 mV dec^−1^ (Fig. 7b), which was much smaller than other electrodes, NF (98.1 mV dec^−1^), pNF (79.3 mV dec^−1^), Ni-MOF/NF (76.6 mV dec^−1^) NiC/NF (39.3 mV dec^−1^) and Ni_2_P@NiC/NF (31.3 mV dec^−1^). The results indicated that the Ni_2_P@Ni-MOF/NF electrode had faster HER kinetics than other electrodes. Since the Tafel slope was smaller than 80 mV dec^−1^, Heyrovsky step was suggested to be the rate-determining step of HER with Ni_2_P@Ni-MOF/NF electrode^61^.

The Nyquist plots of various electrodes for HER are presented in Fig. 7c. The fitting parameters of impedance spectra of HER are summarized in Table S4. It can be clearly seen that the charge transfer resistance of the electrodes follows the same order as that for UOR. The results evidently confirmed that direct phosphidation process could significantly boost the conductivity of Ni-MOF. The electrochemical stability of Ni_2_P@Ni-MOF/NF electrode for HER was further tested by chronoamperometry at − 0.1 V (vs RHE) for 10 h. As shown in Fig. 7d, the current density gradually increased from − 25 to − 20 mA cm^−2^ in the first hour, which may be attributed to the slowly buildup of hydrogen gases bubbles on the surface of the electrode. At the end of the stability test, the current density still remained about 79.3% of the initial value. No obvious deterioration was observed for the LSV curves before and after stability test (Fig. 7e), substantiated the excellent durability of the electrode^62–65^.

### Urea electrolysis

**Figure 8:**
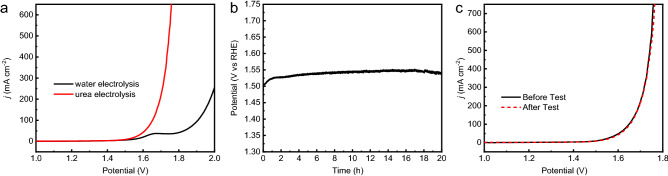
(**a**) LSV polarization curve of urea electrolyzed by Ni_2_P@Ni-MOF/NF electrode in 1 M NaOH with or without 0.33 M urea. (**b**) Chronopotentiometric (V-t) curve of Ni_2_P@Ni-MOF/NF // Ni_2_P@Ni-MOF/NF for urea comprehensive electrolysis at current density of 10 mA cm^-2^. (**c**) LSV curve of Ni_2_P@Ni-MOF/NF before and after 20 h UOR test.

Given the fact that Ni_2_P@Ni-MOF/NF electrode has excellent performance for both UOR and HER, we constructed an electrolyzer with Ni_2_P@Ni-MOF/NF as bifunctional electrode for urea assisted hydrogen production. As shown in Fig. 8a, the electrolyzer could deliver a current density of 100 mA cm^−2^ at 1.65 V for urea electrolysis. While a much higher potential of 1.91 V was required to drive water electrolysis at the same current density. The result clearly demonstrated the advantage of urea electrolysis over water electrolysis for hydrogen production. Compared with other reported electrodes, the Ni_2_P@Ni-MOF/NF was among the best ones for urea electrolysis (Table S3).

The long-term stability for urea electrolysis with Ni_2_P@Ni-MOF/NF electrode at a current density of 10 mA cm^−2^ by chronopotentiometry (V-t). In Fig. 8b, the voltage increase in the first hour was attributed to the buildup of hydrogen gas bubbles on the surface of the cathode, consistent with that observed in Fig. 7d. At the end of the 20-h longevity test, the applied potential increased by only 1.1%, demonstrating the excellent longevity of the sample as bifunctional electrode for urea electrolysis. No appreciable change can be observed for the LSV curves obtained in 2-electrode configuration before and after stability test (Fig. 8c), advocating the excellent stability of the electrode. The results showed that Ni_2_P@Ni-MOF/NF was a promising bifunctional electrode for urea electrolysis.

## Conclusions

In summary, Ni_2_P@Ni-MOF nanosheets were successfully grown on nickel foam through direct phosphidation of Ni-MOF nanosheets. The Ni-MOF structure was partially preserved as confirmed by XRD, TGA and FTIR characterization. The self-supporting Ni_2_P@Ni-MOF/NF exhibited excellent electrochemical performance for both UOR and HER. It only required 1.41 V and 66 mV (vs RHE) to deliver a current density of 100 mA cm^−2^ for UOR and 10 mA cm^−2^ for HER, respectively. The excellent UOR and HER performances of the Ni_2_P@Ni-MOF/NF were attributed to both the enhanced conductivity and the fast release of the gases bubbles from the surfaces of the electrode. The electrolyzer constructed with Ni_2_P@Ni-MOF/NF as both anode and cathode could deliver a current density of 100 mA cm^−2^ in 1 M NaOH with the presence of 0.33 M urea at 1.65 V, which was 0.26 V lower than water electrolysis. Furthermore, the Ni_2_P@Ni-MOF/NF also demonstrated excellent longevity for urea electrolysis. Considering the low cost, easy preparation, long term stability and excel activity, Ni_2_P@Ni-MOF/NF electrode could be a promising bifunctional electrode for hydrogen production through urea electrolysis and to retrieve energy from urea-rich wastewater.

## Methods

### Chemicals and materials

Nickel chloride hexahydrate (NiCl_2_·6H_2_O), N, N-dimethylmethanamide (C_4_H_9_NO, DMF), 1,4-benzenedicarboxylic acid (C_7_H_6_O_2_, BDC), ethanol (C_2_H_6_O), acetone (C_3_H_6_O), urea (CH_4_N_2_O), sodium hydroxide (NaOH) and sodium hypophosphite (NaH_2_PO_2_) were bought from Aladdin (Shanghai, China). Nickel foam (NF) was purchased from Shenzhen Green Creative Environment Technology Co. Ltd. (Shenzhen, China). All of the chemicals and materials were used as received without further purification. Ultrapure water generated using a Ulupure system was used throughout all experiments.

### Synthesis of Ni-MOF/NF

Typically, 0.188 mmol NiCl_2_·6H_2_O and 0.375 mmol BDC were dissolved in a mixture solvent containing 16 mL DMF, 1 mL ethanol and 1 mL water to form a clear solution. The NF was cut into small pieces (2 cm × 3 cm) and sonicated in 2 M HCl for 15 min to remove surface oxides. After washed with copious water and blown dry with pure nitrogen gas, it was then transferred into a Teflon-lined hydrothermal reactor with the above-mentioned solution. Subsequently, the hydrothermal reactor was heated in an oven at 140 °C for 48 h to grow Ni-MOF on NF. Finally, the sample was rinsed with ethanol and ultrapure water thoroughly and dried in air for later use.

### Synthesis of Ni_2_P@Ni-MOF/NF

To synthesis Ni_2_P@Ni-MOF/NF, the Ni-MOF/NF and NaH_2_PO_2_ with a mass ratio of 1:4 were placed on both sides of a porcelain boat. The porcelain boat was then put in a quartz tubing that housed in a tube furnace. The NaH_2_PO_2_ was placed on the upstream of the gas flow. Afterwards, the phosphidation process was carried out for two hours at 300 ℃ under Ar flow. The sample was labelled as Ni_2_P@Ni-MOF/NF. For comparison, the Ni-MOF was fist calcined at 600 ℃ for 2 h under the protection of Ar to get NiC/NF, which was subsequently phosphidized using the same procedure to obtain Ni_2_P@NiC/NF. Direct phosphidation of NF was also performed using the same procedure and the electrode was labelled as pNF.

### Characterization

The morphology of the material was characterized by using scanning electron microscopy (SEM, TESCAN MIRA 3, Czech) equipped with an energy-dispersive X-ray spectrometer (EDX) and transmission electron microscope (HR-TEM, JEM-2010, Japan). X-ray diffraction patterns (XRD, Ulitma IV, Japan) were obtained on a PANalytical XPert instrument with Cu Kα radiation (λ = 0.1542 nm). X-ray photoelectron spectroscopy (XPS, Thermo ESCALAB 250XI, America) was used to study the composition and chemical state of the samples using an Al Kα X-ray source, and the binding energy was calibrated according to the reference C 1s peak at 284.6 eV.

### Electrochemical measurements

Except the test with the electrolyzer, all electrochemical measurements were performed in standard three-electrode configuration on a CHI 760E potentiostat with Ag/AgCl and graphite rod used as reference and counter electrode, respectively. The potentials were reported against reversible hydrogen electrode (RHE) scale by converting the measured potential using the following equation,

2 $$ E_{{{\text{RHE}}}} = E_{{\text{Ag/AgCl}}} + 0.0{59} \times {\text{pH}} + E^{^\circ }_{{\text{Ag/AgCl}}} $$

Electrochemical impedance spectra (EIS) were recorded in the frequency range of 100 kHz to 0.1 Hz at a voltage amplitude of 5 mV. The EIS spectra were fitted to obtain the charge transfer resistance, R_ct_. The electrochemical double-layer capacitance (C_dl_) of electrodes were evaluated by using cyclic voltammetry at scan rates of 20, 40, 60, 80, 100 and 120 mV s^−1^ in a non-Faradic potential range.

## Supplementary Information

Supplementary Information.
