# Supplementary material for: Ni2P nanocrystals embedded Ni-MOF nanosheets supported on nickel foam as bifunctional electrocatalyst for urea electrolysis
Source: Sci Rep. 2021 Nov 1;11:21414. doi: 10.1038/s41598-021-00776-8 (PMC8560839; doi:10.1038/s41598-021-00776-8)
Supplement: Supplementary file 1 — Supplementary Information. [file 41598_2021_776_MOESM1_ESM.docx]

**Supporting Information**

**Ni_2_P nanocrystals embedded Ni-MOF nanosheets supported on nickel foam as Bifunctional Electrocatalyst for Urea Electrolysis**

*Haitao Wang^a^, Haiyan Zou^a^, Yingying Liu^a^, Zhenglong Liu^a^, Wenshuang Sun^a^, Kunyi Andrew Lin^b^, Tielong Li^a^*, Shuangjiang Luo^c^**

^a^MOE Key Laboratory of Pollution Processes and Environmental Criteria, College of Environmental Science and Engineering, Nankai University, Tianjin 300350, P. R China

^b^*Department of Environmental Engineering & Innovation and Development Center of Sustainable Agriculture & Research Center of Sustainable Energy and Nanotechnology, National Chung Hsing University, 250 Kuo-Kuang Road, Taichung, Taiwan, China*

*^c^* Institute of Process Engineering, Chinese Academy of Sciences, Beijing, 100190, China

*Corresponding author: T. Li (litielong@nankai.edu.cn), S. Luo (sjluo@ipe.ac.cn)

**Figure s1.** The TGA curve of Ni-MOF under Ar.

**Figure s2.** The FT-IR spectra of the Ni-MOF before and after phosphidation.


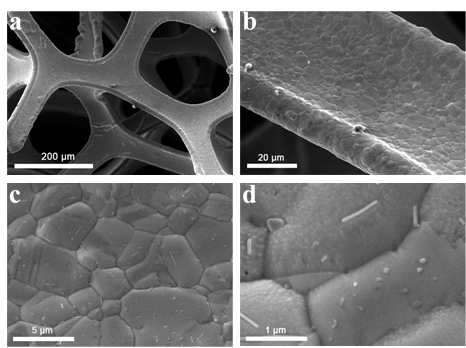


**Figure s3.** SEM image of (a, b) Ni foam and (c, d) pNF.


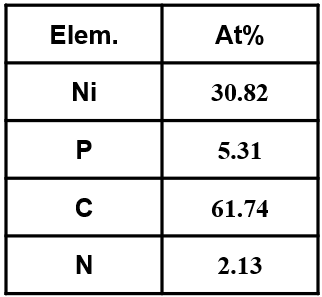


**Figure s4**. Energy-dispersive X-ray (EDX) spectrum of Ni_2_P@Ni-MOF/NF.

| **Electrocatalysts** | **Electrolytes** | **j (mA cm^-2^)** | **Potential (V)** | **Ref.** |
| --- | --- | --- | --- | --- |
| **Ni_2_P/Ni-MOF@NF** | **1 M NaOH**  **+ 0.33 M urea** | **100** | **1.41** | **This work** |
| Ni-MOF@NiO/Ni | 1 M KOH  + 0.33 M urea | 10 | 1.40 | [1] |
| FQD/CoNi-LDH/NF | 1 M KOH  + 0.5 M urea | 100 | 1.42 | [2] |
| MS-Ni_2_P/Ni_0.96_S/NF | 1 M KOH  + 0.5 M urea | 100 | 1.441 | [3] |
| Ni_3_N/Ni_0.2_Mo_0.8_N/NF | 1 M KOH  + 0.5 M urea | 100 | 1.639 | [4] |
| CoS_x_/Co-MOF | 1 M KOH  + 0.5 M urea | 100 | 1.43 | [5] |
| CoRu-MOF/NF | 1 M KOH  + 0.5 M urea | 50 | 1.412 | [6] |
| Se-Ni(OH)_2_@NiSe/NF | 1 M KOH  + 0.33 M urea | 100 | around 1.42 | [7] |
| CoN NF/NF | 1 M KOH  + 0.5 M urea | 100 | around 1.41 | [8] |
| NiO-Ni/NF | 1 M KOH  + 0.33 M urea | 100 | around 1.46 | [9] |
| CoMoO_4_/Co_9_S_8_/NF | 1 M KOH  + 0.5 M urea | 100 | around 1.50 | [10] |

**Table S1.** Comparing Ni_2_P/Ni-MOF@NF with reported electrocatalysts for UOR.


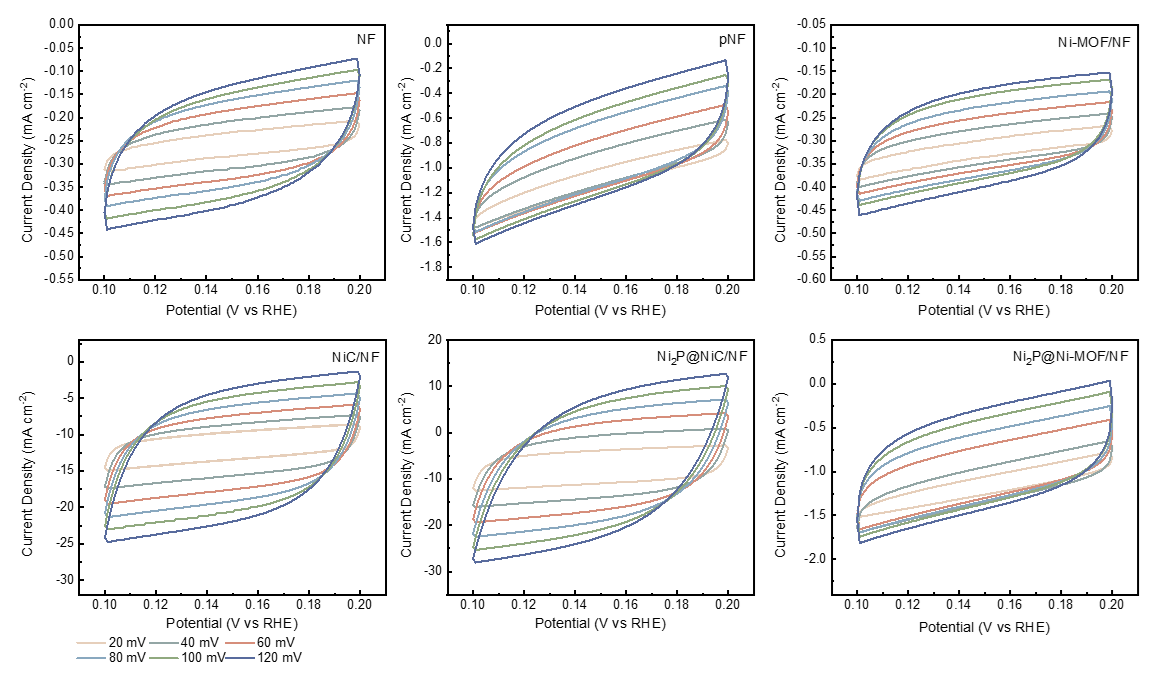


**Figure s5**. CV curves collected at scanning rates from 20 to 120 mV/s for various electrodes.


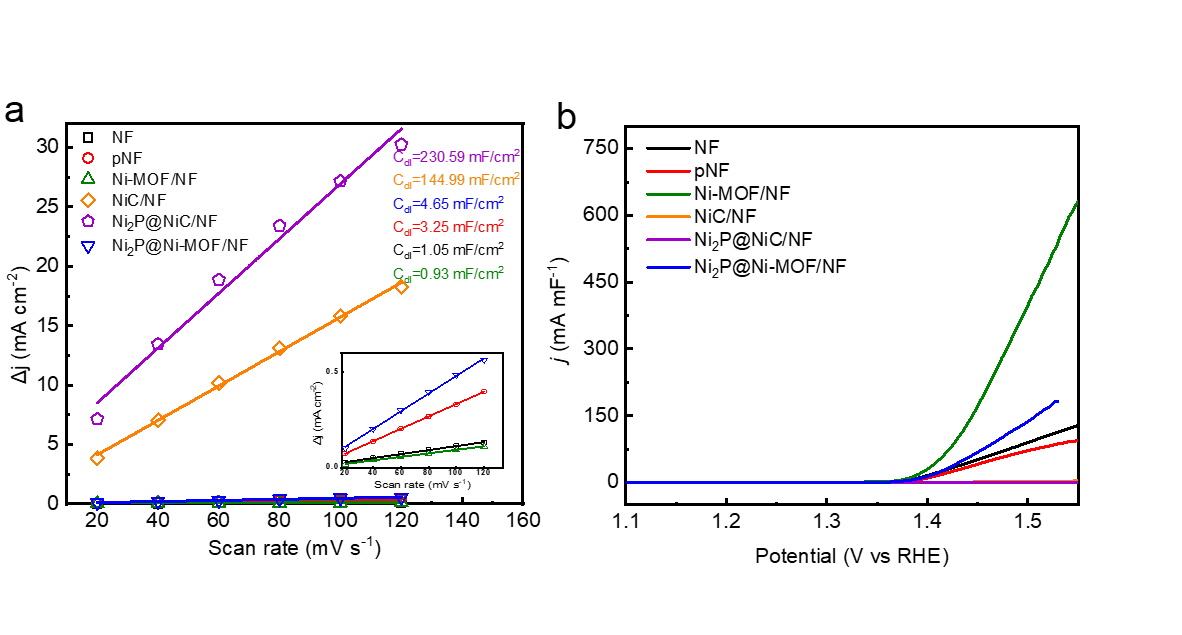


**Figure s6**. (a) Scan rate dependence of the current at potential of 0.15 V vs. Ag/AgCl for various electrodes, (b) LSV curves of various electrode for UOR with current density normalized to C_dl_.


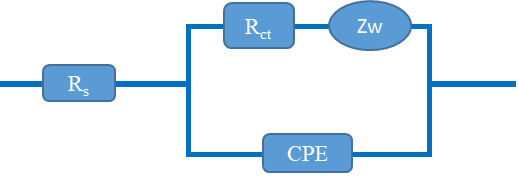


**Figure s7.** Equivalent circuit model for fitting electrochemical impedance spectra of UOR and HER.

The analysis tool Z-fit included in the Correst software was used to select the appropriate equivalent electrical circuit by fitting the impedance spectra. The best fitting was obtained using the equivalent circuit shown in Figure s7. The charge transfer resistance (Rct) is in series with the Warburg impedance (Zw). Rs and CPE are electrolyte resistance and constant phase element, respectively.


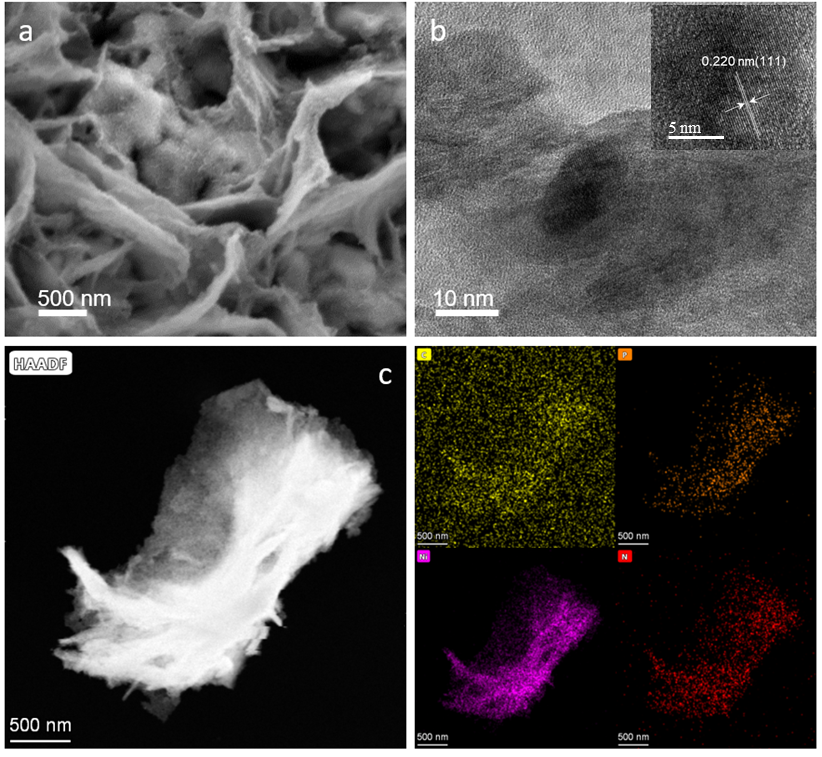


**Figure s8.** (a) SEM image, (b) TEM image and (c) elemental mapping of Ni_2_P@Ni-MOF after stability test.

**Table s2** Parameters of impedance analysis for UOR.

| **Sample** | **Rs/Ω** | **Rct/Ω** | **C_dl_/mF cm^-2^** |
| --- | --- | --- | --- |
| NF | 0.15 | 6.24 | 0.76 |
| pNF | 0.14 | 2.61 | 39.3 |
| Ni-MOF/NF | 0.16 | 2.31 | 46.4 |
| NiC/NF | 0.15 | 0.78 | 112.6 |
| Ni_2_P@NiC/NF | 0.14 | 0.52 | 135.2 |
| Ni_2_P@Ni-MOF/NF | 0.15 | 1.23 | 94.3 |

**Table S3.** Comparing the η_10_ of Ni_2_P/Ni-MOF@NF with literature data.

| **Electrocatalysts** | **Electrolytes** | **substrate** | **η_10_ (mV)** | **Ref.** |
| --- | --- | --- | --- | --- |
| **Ni_2_P/Ni-MOF@NF** | **1 M NaOH** | **NF** | **66** | **This work** |
| Ru-Co_2_P/N-C/NF | 1 M KOH | NF | 65 | [6] |
| Ni_2_P/Ni/NF | 1 M KOH | NF | 98 | [11] |
| MFN-MOFs/NF | 1 M KOH | NF | 79 | [12] |
| NiFe-MOF | 1 M KOH | NF | 134 | [13] |
| CoFePO@NF | 1 M KOH | NF | 87.5 | [14] |
| NiFe/NiCo_2_O4/NF | 1 M KOH | NF | 105 | [15] |
| Ni_0.85_Se/rGO | 1 M KOH | carbon | 128 | [16] |
| N-Ni_3_S_2_/NF | 1 M KOH | NF | 110 | [17] |
| FQD/CoNi-LDH/NF | 1 M KOH | NF | 150 | [2] |
| Ni_2_P@NC/NF | 1 M KOH | NF | 84 | [18] |
| NiCoP-CoP/NF | 1 M KOH | NF | 73 | [19] |
| Ni_2_P-UNMs/NF | 1 M KOH | NF | 75 | [20] |
| Ni_x_P-400 | 1 M KOH | NF | 71 | [21] |
| NiCo_2_S_4_/Ni_3_S_2_/NF | 1 M KOH | NF | 119 | [22] |
| CoP_x_@CNS | 1 M KOH | NF | 91 | [23] |
| FeNi(BDC)(DMF,F)/NF | 1 M KOH | NF | 160 | [24] |

**Table s4** Parameters of impedance analysis for HER.

| **Sample** | **Rs/Ω** | **Rct/Ω** | **C_dl_/mF cm^-2^** |
| --- | --- | --- | --- |
| NF | 2.6 | 121 | 1021 |
| pNF | 2.5 | 63 | 2764 |
| Ni-MOF/NF | 2.6 | 96 | 2986 |
| NiC/NF | 2.6 | 36 | 3606 |
| Ni_2_P@NiC/NF | 2.7 | 26 | 3892 |
| Ni_2_P@Ni-MOF/NF | 2.6 | 34 | 3654 |

**Table s5.** Comparing Ni_2_P/Ni-MOF@NF with other reported bifuntional electrodes for urea electrolysis.

| **Electrode** | **Electrolytes** | V_10_ | **Ref.** |
| --- | --- | --- | --- |
| Ni_2_P/Ni-MOF@NF | 1 M NaOH + 0.33 M urea | 1.50 | **This work** |
| MnO_2_/MnCo_2_O_4_/Ni | 1 M KOH + 0.5 M urea | 1.58 | [25] |
| Co_3_O_4_/NF | 1 M KOH + 0.5 M urea | 1.50 | [26] |
| MNPBA-P | 1 M KOH + 0.5 M urea | 1.50 | [27] |
| FQD/CoNi-LDH/NF | 1 M KOH + 0.5 M urea | 1.59 | [8] |
| Ni-MOF-0.5/NF | 1 M KOH + 0.5 M urea | 1.52 | [28] |

**References**

[1] Q. Li, S. Zheng, M. Du, H. Pang, Ultrathin nanosheet metal–organic framework@NiO/Ni nanorod composites, Chemical Engineering Journal, 417 (2021) 129201.

[2] Y. Feng, X. Wang, J. Huang, P. Dong, J. Ji, J. Li, L. Cao, L. Feng, P. Jin, C. Wang, Decorating CoNi layered double hydroxides nanosheet arrays with fullerene quantum dot anchored on Ni foam for efficient electrocatalytic water splitting and urea electrolysis, Chemical Engineering Journal, 390 (2020) 124525.

[3] M. He, C. Feng, T. Liao, S. Hu, H. Wu, Z. Sun, Low-Cost Ni_2_P/Ni_0.96_S Heterostructured Bifunctional Electrocatalyst toward Highly Efficient Overall Urea-Water Electrolysis, ACS Applied Materials & Interfaces, 12 (2020) 2225-2233.

[4] R.-Q. Li, X.-Y. Wan, B.-L. Chen, R.-Y. Cao, Q.-H. Ji, J. Deng, K.-G. Qu, X.-B. Wang, Y.-C. Zhu, Hierarchical Ni_3_N/Ni_0.2_Mo_0.8_N heterostructure nanorods arrays as efficient electrocatalysts for overall water and urea electrolysis, Chemical Engineering Journal, 409 (2021) 128240.

[5] H. Xu, K. Ye, K. Zhu, J. Yin, J. Yan, G. Wang, D. Cao, Template-directed assembly of urchin-like CoS_x_/Co-MOF as an efficient bifunctional electrocatalyst for overall water and urea electrolysis, Inorganic Chemistry Frontiers, 7 (2020) 2602-2610.

[6] Y. Xu, T. Ren, K. Ren, S. Yu, M. Liu, Z. Wang, X. Li, L. Wang, H. Wang, Metal-organic frameworks-derived Ru-doped Co_2_P/N-doped carbon composite nanosheet arrays as bifunctional electrocatalysts for hydrogen evolution and urea oxidation, Chemical Engineering Journal, 408 (2021) 127308.

[7] C. Tang, Z.L. Zhao, J. Chen, B. Li, L. Chen, C.M. Li, Se-Ni(OH)_2_-shelled vertically oriented NiSe nanowires as a superior electrocatalyst toward urea oxidation reaction of fuel cells, Electrochimica Acta, 248 (2017) 243-249.

[8] Y. Chen, P. Sun, W. Xing, Cobalt nitride nanoflakes supported on Ni foam as a high-performance bifunctional catalyst for hydrogen production via urea electrolysis, Journal of Chemical Sciences, 131 (2019) 101.

[9] Z. Yue, W. Zhu, Y. Li, Z. Wei, N. Hu, Y. Suo, J. Wang, Surface Engineering of a Nickel Oxide–Nickel Hybrid Nanoarray as a Versatile Catalyst for Both Superior Water and Urea Oxidation, Inorganic Chemistry, 57 (2018) 4693-4698.

[10] X. Du, C. Huang, X. Zhang, Synthesis of CoMoO_4_/Co_9_S_8_ network arrays on nickel foam as efficient urea oxidation and hydrogen evolution catalyst, International Journal of Hydrogen Energy, 44 (2019) 19595-19602.

[11] B. You, N. Jiang, M. Sheng, M.W. Bhushan, Y. Sun, Hierarchically Porous Urchin-Like Ni_2_P Superstructures Supported on Nickel Foam as Efficient Bifunctional Electrocatalysts for Overall Water Splitting, ACS Catalysis, 6 (2016) 714-721.

[12] D. Senthil Raja, H.-W. Lin, S.-Y. Lu, Synergistically well-mixed MOFs grown on nickel foam as highly efficient durable bifunctional electrocatalysts for overall water splitting at high current densities, Nano Energy, 57 (2019) 1-13.

[13] J. Duan, S. Chen, C. Zhao, Ultrathin metal-organic framework array for efficient electrocatalytic water splitting, Nature Communications, 8 (2017) 15341.

[14] J. Duan, S. Chen, A. Vasileff, S.Z. Qiao, Anion and Cation Modulation in Metal Compounds for Bifunctional Overall Water Splitting, ACS Nano, 10 (2016) 8738-8745.

[15] C. Xiao, Y. Li, X. Lu, C. Zhao, Bifunctional Porous NiFe/NiCo_2_O_4_/Ni Foam Electrodes with Triple Hierarchy and Double Synergies for Efficient Whole Cell Water Splitting, Advanced Functional Materials, 26 (2016) 3515-3523.

[16] Min Zhu, Yongde Yan, Qing Yan, Jinling Yin, Guiling Wang. In situ growth of Ni0·85Se on graphene as a robust electrocatalyst for hydrogen evolution reaction. International Journal of Hydrogen Energy, 45 (2019) 10486-10493.

[17] P. Chen, T. Zhou, M. Zhang, Y. Tong, C. Zhong, N. Zhang, L. Zhang, C. Wu, Y. Xie, 3D Nitrogen-Anion-Decorated Nickel Sulfides for Highly Efficient Overall Water Splitting, Advanced Materials, 29 (2017) 1701584.

[18] X.-W. Lv, Z.-P. Hu, L. Chen, J.-T. Ren, Y.-P. Liu, Z.-Y. Yuan, Organic–Inorganic Metal Phosphonate-Derived Nitrogen-Doped Core–Shell Ni_2_P Nanoparticles Supported on Ni Foam for Efficient Hydrogen Evolution Reaction at All pH Values, ACS Sustainable Chemistry & Engineering, 7 (2019) 12770-12778.

[19] H. Liu, X. Ma, H. Hu, Y. Pan, W. Zhao, J. Liu, X. Zhao, J. Wang, Z. Yang, Q. Zhao, H. Ning, M. Wu, Robust NiCoP/CoP Heterostructures for Highly Efficient Hydrogen Evolution Electrocatalysis in Alkaline Solution, ACS Applied Materials & Interfaces, 11 (2019) 15528-15536.

[20] Y. Ding, B.-Q. Miao, S.-N. Li, Y.-C. Jiang, Y.-Y. Liu, H.-C. Yao, Y. Chen, Benzylamine oxidation boosted electrochemical water-splitting: Hydrogen and benzonitrile co-production at ultra-thin Ni_2_P nanomeshes grown on nickel foam, Applied Catalysis B: Environmental, 268 (2020) 118393.

[21] Y. Wang, L. Liu, X. Zhang, F. Yan, C. Zhu, Y. Chen, Self-supported tripod-like nickel phosphide nanowire arrays for hydrogen evolution, Journal of Materials Chemistry A, 7 (2019) 22412-22419.

[22] H. Liu, X. Ma, Y. Rao, Y. Liu, J. Liu, L. Wang, M. Wu, Heteromorphic NiCo_2_S_4_/Ni_3_S_2_/Ni Foam as a Self-Standing Electrode for Hydrogen Evolution Reaction in Alkaline Solution, ACS Applied Materials & Interfaces, 10 (2018) 10890-10897.

[23] C.-C. Hou, L. Zou, Y. Wang, Q. Xu, MOF-Mediated Fabrication of a Porous 3D Superstructure of Carbon Nanosheets Decorated with Ultrafine Cobalt Phosphide Nanoparticles for Efficient Electrocatalysis and Zinc–Air Batteries, Angewandte Chemie International Edition, 59 (2020) 21360-21366.

[24] H.-W. Lin, D. Senthil Raja, X.-F. Chuah, C.-T. Hsieh, Y.-A. Chen, S.-Y. Lu, Bi-metallic MOFs possessing hierarchical synergistic effects as high performance electrocatalysts for overall water splitting at high current densities, Applied Catalysis B: Environmental, 258 (2019) 118023.

[25] C. Xiao, S. Li, X. Zhang, D.R. MacFarlane, MnO_2_/MnCo_2_O_4_/Ni heterostructure with quadruple hierarchy: a bifunctional electrode architecture for overall urea oxidation, Journal of Materials Chemistry A, 5 (2017) 7825-7832.

[26] X. Du, C. Huang, X. Zhang, Co_3_O_4_ arrays with tailored morphology as robust water oxidation and urea splitting catalyst, Journal of Alloys and Compounds, 809 (2019) 151821.

[27] H. Xu, K. Ye, K. Zhu, Y. Gao, J. Yin, J. Yan, G. Wang, D. Cao, Transforming Carnation-Shaped MOF-Ni to Ni–Fe Prussian Blue Analogue Derived Efficient Bifunctional Electrocatalyst for Urea Electrolysis, ACS Sustainable Chemistry & Engineering, 8 (2020) 16037-16045.

[28] S. Zheng, Y. Zheng, H. Xue, H. Pang, Ultrathin nickel terephthalate nanosheet three-dimensional aggregates with disordered layers for highly efficient overall urea electrolysis, Chemical Engineering Journal, 395 (2020) 125166.
